# Evaluation of mobile real-time polymerase chain reaction tests for the detection of severe acute respiratory syndrome coronavirus 2

**DOI:** 10.1038/s41598-021-88625-6

**Published:** 2021-04-30

**Authors:** Chukwunonso Onyilagha, Henna Mistry, Peter Marszal, Mathieu Pinette, Darwyn Kobasa, Nikesh Tailor, Yohannes Berhane, Charles Nfon, Bradley Pickering, Samira Mubareka, David Bulir, Sylvia Chong, Robert Kozak, Aruna Ambagala

**Affiliations:** 1 National Centre for Foreign Animal Disease, Canadian Food Inspection Agency, Winnipeg, MB Canada; 2 Sunnybrook Health Sciences Centre, Toronto, ON Canada; 3 St. Joseph’s Healthcare, Hamilton, ON Canada; 4 National Microbiology Laboratory, Public Health Agency of Canada, Winnipeg, Canada; 5 Department of Comparative Biology, Faculty of Veterinary Medicine, University of Calgary, Calgary, AB Canada; 6 Department of Medical Microbiology and Infectious Diseases, University of Manitoba, Winnipeg, MB Canada

**Keywords:** Diagnosis, Viral infection, Microbiology, Molecular biology

## Abstract

The coronavirus disease 2019 (COVID-19) pandemic, caused by severe acute respiratory syndrome coronavirus 2 (SARS-CoV-2), calls for prompt and accurate diagnosis and rapid turnaround time for test results to limit transmission. Here, we evaluated two independent molecular assays, the Biomeme SARS-CoV-2 test, and the Precision Biomonitoring TripleLock SARS-CoV-2 test on a field-deployable point-of-care real-time PCR instrument, Franklin three9, in combination with Biomeme M1 Sample Prep Cartridge Kit for RNA 2.0 (M1) manual extraction system for rapid, specific, and sensitive detection of SARS-COV-2 in cell culture, human, and animal clinical samples. The Biomeme SARS-CoV-2 assay, which simultaneously detects two viral targets, the *orf1ab* and S genes, and the Precision Biomonitoring TripleLock SARS-CoV-2 assay that targets the 5′ untranslated region (5′ UTR) and the envelope (E) gene of SARS-CoV-2 were highly sensitive and detected as low as 15 SARS-CoV-2 genome copies per reaction. In addition, the two assays were specific and showed no cross-reactivity with Middle Eastern respiratory syndrome coronavirus (MERS-CoV), infectious bronchitis virus (IBV), porcine epidemic diarrhea virus (PEDV), transmissible gastroenteritis (TGE) virus, and other common human respiratory viruses and bacterial pathogens. Also, both assays were highly reproducible across different operators and instruments. When used to test animal samples, both assays equally detected SARS-CoV-2 genetic materials in the swabs from SARS-CoV-2-infected hamsters. The M1 lysis buffer completely inactivated SARS-CoV-2 within 10 min at room temperature enabling safe handling of clinical samples. Collectively, these results show that the Biomeme and Precision Biomonitoring TripleLock SARS-CoV-2 mobile testing platforms could reliably and promptly detect SARS-CoV-2 in both human and animal clinical samples in approximately an hour and can be used in remote areas or health care settings not traditionally serviced by a microbiology laboratory.

## Introduction

To date, over 128 million people living in 223 countries and territories have contracted SARS-CoV-2, with more than 2.8 million deaths reported worldwide^1^. Due to the highly infectious nature of SARS-CoV-2 there is a need for rapid and early diagnosis of the disease to help with the early implementation of mitigation practices. Molecular diagnostic testing is one of the WHO-recommended, quickest, and most sensitive ways of testing for SARS-CoV-2 in patient samples^2^. However, one of the challenges faced by health workers, in remote or resource-limited settings, is the requirement to transport patient samples to the central laboratories for testing and confirmation of cases, which causes a significant increase in the test result turnaround time. A reliably quicker way of testing for COVID-19, without the need to transport samples to centralized microbiology laboratories, would significantly improve the result turnaround time.

The Biomeme SARS-CoV-2 and Precision Biomonitoring TripleLock SARS-CoV-2 assays, when used in combination with the M1 manual extraction kit, are designed to quickly and reliably detect SARS-CoV-2. These tests are triplex real-time-PCR-based assays, with one target serving as a process control. They come in strips of tubes containing fully lyophilized primers, probes, and master mix (enzymes) that require only the addition of nucleic acid to run a test. The Biomeme SARS-CoV-2 test allows for the specific detection of SARS-CoV-2 Open Reading Frame 1ab (Orf1ab) and Spike (S) genes, while an RNA Process Control spiked into the sample (RNA extraction and RT-PCR control utilizing MS2 bacteriophage), similar to RNaseP below, must be detected for negative SARS-CoV-2 result to be valid. The Orf1ab encodes replicase polyproteins, which are essential for the successful replication and transcription of viral RNA^3^. In contrast, the S gene encodes proteins responsible for viral attachment to host receptors^4^. Comparatively, the Precision Biomonitoring TripleLock SARS-CoV-2 test was designed to detect the 5′ untranslated region (5′ UTR) and the envelope (E) gene of the SARS-CoV-2. The 5′ UTR contains stem-loop structures that play a functional role in viral replication, whereas the E gene is involved in virus assembly, budding, and pathogenesis^5,6^. This test uses human RNaseP, a ubiquitously expressed gene in all human cells^7^, as the third target to serve as an internal control for RNA extraction and PCR amplification. This study describes both Biomeme SARS-CoV-2 and the Precision Biomonitoring TripleLock SARS-CoV-2 tests on Franklin three9, a portable real-time PCR instrument that has been used to successfully detect foot‐and‐mouth disease^8^ and African swine fever^9^ viruses in clinical samples. Here we describe the performance characteristics of each assay using clinical and animal samples and demonstrate the potential applications for settings outside of a traditional microbiology laboratory.

## Materials and methods

### Ethics statement

Patient samples used in this study were collected as part of routine clinical testing. Residual sample material from de-identified patients was used for evaluation of the assays and was determined to be quality improvement initiative and ethics approval was not required. The animal experiments in this study were performed in accordance with the guidelines issued by the Canadian Council on Animal Care, under the approval of the Animal Care Committee at the Canadian Science Centre for Human and Animal Health.

### Inactivation of SARS-CoV-2 by M1 lysis buffer

The SARS-CoV-2 (P3) isolate (hCoV-19/Canada/ON-VIDO-01/2020, GISAID accession # EPI_ISL_425177), which was used throughout the study for both assays, was propagated in Vero E6 cells inside the Biosafety Level 3 plus (BSL-3 +) laboratory at the National Centre for Foreign Animal Disease (NCFAD). To determine the ability of M1 lysis buffer to inactivate SARS-CoV-2, 200 µl of the lysis buffer was mixed with 50 µl of cell culture-amplified SARS-CoV-2 (3 × 10^6^ pfu/ml) and incubated for 10 min at room temperature with occasional mixing. The lysis buffer-treated virus mixture was then diluted 200 times by adding 50 ml of Dulbecco’s Modified Eagle Medium (DMEM, Thermo Fisher Scientific, Mississauga, ON) containing 2% fetal bovine serum (FBS, Thermo Fisher Scientific, Mississauga, ON). Five T75 flasks (Thermo Fisher Scientific, Mississauga, ON) of Vero E6 cells were inoculated (10 ml per flask) with the lysis buffer-treated virus. Separate T75 flasks were either inoculated with 10 µl of the virus in 10 ml of DMEM containing 2% FBS (positive control) or 10 ml DMEM containing 2% FBS (negative control) and incubated at 37 °C for three days. After three days of incubation, the SARS-CoV-2-induced cytopathic effect (CPE) was assessed, and the flasks were frozen at − 70 °C. Forty-eight hours later, flasks were thawed, and the contents were clarified by centrifugation at 2000×*g* for 15 min. The supernatant from each of the flasks was collected and used to inoculate fresh Vero E6 cells (10 ml per flask). After 1-h incubation, 10 ml of DMEM containing 4% FBS was added to each flask before returning them to the incubator. On days 3 and 6 post-inoculation, CPE was reassessed in each flask, and the results were recorded.

### Nucleic acid extraction and real-time reverse transcription polymerase chain reaction (RRT-PCR) platforms

Cell culture-derived virus was first inactivated in M1 lysis buffer (Biomeme Inc., Philadelphia, PA) in the NCFAD BSL-3 + laboratory. Briefly, 300 µl of cell culture-amplified SARS-CoV-2 (1.1 × 10^6^ pfu/ml) was directly added to 1.5 ml of Biomeme lysis buffer for inactivation and incubated for 10 min at room temperature. The inactivated virus in M1 lysis buffer was then transferred to the NCFAD BSL-3 laboratory for the remaining steps of the M1 extraction (binding, protein wash, salt wash, drying wash, air drying, and elution of pure nucleic acids). For the SARS-CoV-2 E_Sarbeco real-time PCR assay^10^, recommended by the WHO, the nucleic acid was extracted using MagMAX™ CORE Nucleic Acid Purification Kit (CORE kit) according to the manufacturer’s recommendations with slight modification. Briefly, 650 µl of the TriPure-inactivated sample (1:9 ratio), 350 µl of CORE binding buffer (spiked with Armored RNA Enterovirus- internal control, Asuragen), and 30 µl of magnetic bead-proteinase K mixture, were used in the RNA extraction process, which was eluted in 30 µl of elution buffer. Two RRT-PCR assays were evaluated using the same workflow from M1 RNA extraction to RRT-PCR results. The Biomeme RRT- PCR assay targets the Orf1ab (FAM) and S (ATTO647N) genes of SARS-CoV-2, with the spiked RNA Process Control (TexasRedX). The Precision Biomonitoring RRT- PCR assay targets the 5′UTR (FAM) and E gene (TexasRedX) as well as RNaseP (ATTO647N) as internal process control, eliminating the need to spike samples with an extraction and PCR control. All the real-time PCR assays reported were performed on a portable RRT-PCR thermocycler, Franklin three9 (Biomeme Inc., Philadelphia, PA). For both assays, 20 µl of the nucleic acid was added directly to a fully-lyophilized master mix containing the specific primers and probes for the targets. The Biomeme-recommended thermal profile (RT step of 55 °C for 120 s; initial denature step of 95 °C for 60 s, and 45 cycles of 95 °C for 3 s, & 60 °C for 30 s) and the Precision Biomonitoring thermal profile (55 °C for 10 min; initial denature step of 95 °C for 2 min, and 45 cycles of 95 °C for 5 s, & 60 °C for 20 s) were used for the respective tests. Samples with cycle threshold (Ct) value less than 40 for SARS-CoV-2 targets were considered positive, and the samples negative for SARS-CoV-2 genome but positive for RNA Process Control or RNaseP were considered negatives. Also, according to the Biomeme SARS-CoV-2 assay design, the RNA Process Control may not amplify when the two SARS-CoV-2 gene signals are very strong. The SARS-CoV-2 E_Sarbeco real-time PCR was set up with 4X TaqMan^®^ Fast Virus 1-Step master mix (Thermo Fisher Scientific, Mississauga, ON) with the recommended primers, probes, and final reagent concentrations^10^. The reaction was run on the Applied Biosystems 7500™ (Thermo Fisher Scientific, Mississauga, ON) instrument.

### Limit of detection

Relative quantification of the limit of detection (LOD) for the Biomeme and Precision Biomonitoring SARS-CoV-2 assays was determined with target-specific gBlocks™ Gene Fragments (ID, San Diego, CA) using cell culture-derived SARS-CoV-2 sample (15 × 10^8^ copies per reaction). The sample was serially diluted, run through the M1 extraction process as described above, and the extracted nucleic acid was tested with the Biomeme and Precision Biomonitoring TripleLock SARS-CoV-2 assays to assess specificity and LOD.

### Specificity

Cell culture-derived SARS-CoV-1, MERS-CoV, IBV Iowa, pH1N1 virus, PEDV Colorado, and TGEV F-216 were used for specificity testing. The SARS-CoV-1, MERS-CoV, and pH1N1 virus were inactivated in the BSL-3 + laboratory (standard NCFAD procedure) by TriPure Reagent (Roche Canada, Laval, QC) before transfer to BSL-3 laboratory for nucleic acid extraction and real-time PCR assay. For nucleic acid extraction, the M1 (SARS-CoV-1 and MERS-CoV) or MagMAX™ CORE (IBV, pH1N1, PEDV, and TGE, Thermo Fisher Scientific, Mississauga, ON) were used. The nucleic acids extracted were pre-tested with their respective NCFAD real-time PCR assays and confirmed to be positives before being used in the Biomeme SARS-CoV-2 assay (single detection). The viruses used for this assessment were obtained from the NCFAD where they have been characterized (using molecular methods -conventional and/or real-time PCR) and maintained. For the Precision Biomonitoring TripleLock SARS CoV-2 assay, archived patient specimens containing common respiratory pathogens were used. The samples (200 µl each in UTM) previously (2018 and 2019) tested positive by xTAG RVP FAST v2 (Luminex, Toronto, Canada) following M1 extraction. Also, common respiratory bacteria isolated from a healthy volunteer were spiked into nasopharyngeal (NP) UTM swab aliquots and used for M1 extraction followed by SARS CoV-2 testing.

### Reproducibility

For the Biomeme SARS-CoV-2 assay repeatability experiment, eight M1-extracted cell culture-amplified SARS-CoV-2 (all at 10^–4^ dilution) were tested by three technicians from the NCFAD across three Franklin three9 instruments. Each technician ran the samples twice on each Franklin instrument. Similarly, to assess the repeatability of the Precision Biomonitoring TripleLock SARS-CoV-2 assay, five identical samples of cell culture-amplified SARS-CoV-2 extracted on different days using M1 cartridges were tested on three Franklin three9 instruments in five replicates each.

### Clinical evaluation

A mix of 40 known-negative and 50 SARS-CoV-2 known-positive patient nasal swab samples were tested on the Franklin three9 using the Biomeme SARS-CoV-2 assay. Patient samples that were negative for COVID-19 also included ones where other respiratory viruses had been detected by conventional clinical assays. The samples were assessed using the relevant clinical gold-standard test where specimens were either extracted using the NucliSENS EasyMAG and subsequently run on the Rotor-gene Q, detecting the 5′UTR and envelope gene or were extracted and run on the BD Max 5′UTR assay, detecting only the 5′UTR gene^11^. Additionally, negative patient samples were spiked with various clinical bacterial isolates to determine specificity. The clinical evaluation of the Precision Biomonitoring TripleLock SARS-CoV-2 Assay was conducted using 63 known-positive and 64 known-negative patient specimens. These samples were extracted using the NucliSENS EasyMAG kit and were confirmed positive or negative using a modified 2019-nCoV CDC EUA Kit (Integrated DNA Technologies, Coralville, USA) with the Luna Universal Probe One-Step RT kit (New England BioLabs, Whitby, Canada) and SARS-CoV-2 envelope gene assays^11^. Extraction and RRT-PCR setup for the Biomeme and Precision Biomonitoring SARS-CoV-2 assays were carried out as described above for patient specimens.

### Animal sample evaluation

Five to six-week old hamsters were intranasally inoculated with 10^5^ TCID_50_ (in 100 µl Dulbecco’s modified essential medium) SARS-CoV-2 (SARS-CoV-2;hCoV-19/Canada/ON-VIDO492 01/2020, GISAID accession # EPI_ISL_425177), which was isolated from a clinical specimen obtained at the Sunnybrook Research Institute (SRI)/ University of Toronto on VeroE6 cells and provided by the Vaccine and Infectious Disease Organization (VIDO) with permission. The infected animals were sacrificed on days 2 and 5 post-infection and the nasal wash, rectal, and oral swabs were collected and 140 µl of each was used for M1 extraction. For sample testing with the Biomeme and Precision Biomonitoring SARS-CoV-2 assays, 20 µl of the extracted RNA was used to set up the reaction on Franklin three9. The study was carried out in compliance with the ARRIVE guidelines.

### M1 compatibility with alternate swab media

Nasal swabs were most frequently collected in universal transport media (Copan UTM) or viral transport media (VTM), however, liquid Amies (e-Swab), Cobas PCR media (Roche Canada), and Bartels FlexTrans transport medium (Trinity Biotech) are also commonly used in the clinical laboratories. Following M1 extraction, we tested 29 known positive specimens (initially assessed by Rotor gene Q 5′UTR/envelope assay or BD Max 5′UTR assays) collected in the aforementioned alternative swab media (n_E_ = 11, n_C_ = 11, and n_B_ = 7 respectively) on the Franklin three9 using the Precision Biomonitoring TripleLock SARS-CoV-2 assay. The M1 extraction protocol is common to Biomeme and Precision Biomonitoring SARS-CoV-2 assays.

## Results

### The complete inactivation of SARS-CoV-2 by M1 lysis buffer

**Figure 1:**
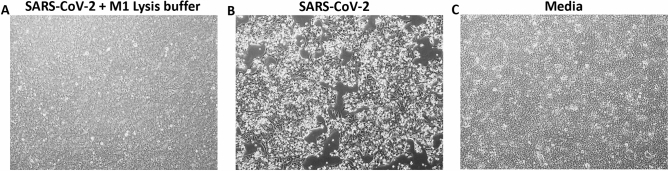
Inactivation of SARS-CoV-2 by M1 lysis buffer. The M1 lysis buffer was incubated with SARS-CoV-2 for 10 min and the mixture was used to inoculate VeroE6 cells in T75 flasks; on day 6 after the second passage, cytopathic effect (CPE) was assessed in the flasks **(A)** as well as in the flasks containing untreated SARS-CoV-2 **(B)** and media **(C)**. **(B)** is a representation of 5 different flasks with similar CPE.

To safely use the Biomeme and Precision Biomonitoring SARS-CoV-2 Test kits at the point-of-care, it is critical to demonstrate a complete inactivation of SARS-CoV-2 by the lysis buffer in the M1 extraction cartridge. Working with inactivated viruses adds to the safety of the health workers by reducing the risk of exposure to live SARS-CoV-2 during testing. To evaluate this, we incubated high-tittered SARS-CoV-2 (3 × 10^6^ pfu/ml) in M1 lysis buffer for 10 min at room temperature and used the lysis buffer-treated virus to inoculate Vero E6 cells. While the untreated SARS-CoV-2 caused CPE on Vero E6 cells, the lysis buffer-treated SARS-CoV-2 or media alone caused no CPE (Fig. 1A–C) even after two passages. This result indicates that the M1 lysis buffer completely inactivated SARS-CoV-2 in the samples.

### Sensitivity and LOD of the SARS-CoV-2 assays

Because clinical COVID-19 samples vary in viral loads, it is crucial to determine the sensitivity of the Biomeme and Precision Biomonitoring SARS-CoV-2 assays. Cell culture-amplified SARS-CoV-2 sample with known copy number (15 × 10^8^ copies per reaction) was serially-diluted, subjected to M1 extraction, and tested with the Biomeme and Precision Biomonitoring TripleLock SARS-CoV-2 assays on Franklin three9. The results show that the Biomeme SARS-CoV-2 assays and Precision Biomonitoring TripleLock SARS-CoV-2 assay are highly sensitive and can detect the viral RNA even in highly diluted samples as low as 15 genomic copies per reaction (Table 1A) assuming no loss during M1 extraction.

**Table 1:** The sensitivity and LOD of the Biomeme and Precision Biomonitoring SARS-CoV-2 tests.

(A)					
SARS-CoV-2	Biomeme SARS-CoV-2 test		SARS-CoV-2 (estimated copies per reaction)	Precision Biomonitoring TripleLock SARS-CoV-2 test	
	Target	Ct value		Target	Ct value
Undiluted	Orf1ab gene	11.24	15 × 10^8^	5′UTR gene	11.40
	S gene	10.13		E gene	10.24
10^–1^	Orf1ab gene	14.31	15 × 10^7^	5′UTR gene	14.40
	S gene	14.48		E gene	12.92
10^–2^	Orf1ab gene	17.29	15 × 10^6^	5′UTR gene	17.68
	S gene	17.45		E gene	16.06
10^–3^	Orf1ab gene	21.31	15 × 10^5^	5′UTR gene	21.01
	S gene	21.48		E gene	19.47
10^–4^	Orf1ab gene	24.24	15 × 10^4^	5′UTR gene	24.28
	S gene	24.43		E gene	22.47
10^–5^	Orf1ab gene	27.96	15 × 10^3^	5′UTR gene	27.76
	S gene	28.12		E gene	26.26
10^–6^	Orf1ab gene	31.39	15 × 10^2^	5′UTR gene	31.39
	S gene	30.99		E gene	29.23
10^–7^	Orf1ab gene	32.65	15 × 10^1^	5′UTR gene	33.45
	S gene	33.99		E gene	32.63
10^–8^	Orf1ab gene	34.69	15 × 10^0^	5′UTR gene	35.76
	S gene	33.82		E gene	34.14
10^–9^	Orf1ab gene	–	15 × 10^–1^	5′UTR gene	–
	S gene	–		E gene	–

(B)					
SARS-CoV-2	Target	Ct value			
		Biomeme	E_Sarbeco		
Undiluted	Orf1ab gene	9.47	13.36		
	S gene	9.84			
10^–1^	Orf1ab gene	12.09	16.47		
	S gene	12.31			
10^–2^	Orf1ab gene	16.73	21.30		
	S gene	16.94			
10^–3^	Orf1ab gene	20.49	25.00		
	S gene	20.61			
10^–4^	Orf1ab gene	24.78	28.66		
	S gene	24.61			
10^–5^	Orf1ab gene	28.36	31.99		
	S gene	27.58			
10^–6^	Orf1ab gene	31.78	34.89		
	S gene	30.87			
10^–7^	Orf1ab gene	32.92	37.14		
	S gene	32.91			
NTC	Orf1ab gene	0.00	0.00		
	S gene	0.00			

The M1 Sample Prep Cartridge Kit for RNA 2.0 was used to extract RNA from serial dilutions of cell culture-amplified SARS-CoV-2. Following the extractions, 20 µl of RNA from each dilution was used to set up the Biomeme and Precision Biomonitoring SARS-CoV-2 Test on Franklin three9 (A). Because the same extracted RNA from the dilutions were used for both assays, there was no spiking of RPC during extraction as required by the Biomeme assay, this was done to avoid interfering with the E gene of the Precision Biomitoring assay as both RPC and E gene are on the same channel (TexasRedX) on Franklin three9; as a result, all internal controls were excluded from the runs and analysis. In addition, the detection of the full SARS-CoV-2 targets (even in the absence of the internal controls) in both assays means that the tests are positive/valid. In another experiment, MagMAX™ CORE Nucleic Acid Purification Kit-extracted RNA from cell culture-amplified SARS-CoV-2 dilutions was used to set up Biomeme and E_Sarbeco SARS-CoV-2 assays to compare their sensitivities. The Biomeme SARS-CoV-2 assay was run on Franklin three9 while the E_Sarbeco assay was run on ABI 7500 (B).

Since Biomeme and Precision Biomonitoring SARS-CoV-2 assays have similar sensitivity, we further compared the assay sensitivity of the representative Biomeme and the E_Sarbeco assay^10^. Although the SARS-CoV-2 targets for both assays are different, results show that the Biomeme SARS-CoV-2 assay is slightly more sensitive by approximately tenfold (based on full target detection in the same sample dilution) than the E_Sarbeco assay (Table 1B).

### Specificity of the SARS-CoV-2 assays

Another essential feature of any diagnostic test is the specificity, both for use in clinical contexts and for identifying animal reservoirs in field studies. The Biomeme SARS-CoV-2 assay was tested against several avian and porcine coronaviruses as well as SARS-CoV-1, MERS-CoV, seasonal human coronaviruses, and other common respiratory pathogens. The assay only detected SARS-CoV-2 genome, but not that of other pathogens tested (Table 2). The Precision Biomonitoring TripleLock SARS-CoV-2 assay did not cross-react with most of the common respiratory viruses or bacterial pathogens and MERS-CoV but detected both SARS-CoV-1 and SARS-CoV-2 genomic material (Table 2). Conclusively, these results show that the Biomeme SARS-CoV-2 test is highly sensitive and specific for SARS-CoV-2. Moreover, as SARS-CoV-1 is not known to be in circulation, there is less clinical concern about the observed assay cross-reactivity in the Precision Biomonitoring TripleLock SARS-CoV-2 test.

**Table 2:** The specificity of the Biomeme and Precision Biomonitoring SARS-CoV-2 tests.

Confirmed pathogen	Biomeme SARS-CoV-2 test			Precision Biomonitoring TripleLock SARS-CoV-2 test		
	Orf1ab gene	S gene	RNA process control	5′UTR gene	E gene	RNaseP (averaged)
Severe acute respiratory syndrome coronavirus 2 (SARS-CoV-2)	12.60	12.78	No Ct*	14.27	11.17	41.12
Severe acute respiratory syndrome coronavirus (SARS-CoV-1)	No Ct	No Ct	26.15	16.38	13.26	29.20
Middle east respiratory syndrome (MERS) virus	No Ct	No Ct	26.44	No Ct	No Ct	30.95
Infectious bronchitis virus (IBV) Iowa	No Ct	No Ct	31.44	–	–	–
Pandemic H1N1 (pH1N1) virus	No Ct	No Ct	29.76	–	–	–
Porcine epidemic diarrhea virus (PEDV) Colorado	No Ct	No Ct	31.98	–	–	–
Transmissible gastroenteritis (TGE) virus F-216	No Ct	No Ct	31.21	–	–	–
Influenza A H3N2	No Ct	No Ct	25.87	No Ct	No Ct	26.29
Influenza A H1N1	No Ct	No Ct	27.11	No Ct	No Ct	28.41
Rhinovirus (ENR/RHV)	No Ct	No Ct	13.34**	No Ct	No Ct	30.40
Human coronavirus OC43 (HCoV-OC43)	No Ct	No Ct	26.56	No Ct	No Ct	27.65
Human coronavirus 229E (HCoV-229E)	No Ct	No Ct	27.62	No Ct	No Ct	23.91
Human coronavirus NL63 (HCoV-NL63)	No Ct	No Ct	26.79	No Ct	No Ct	27.57
Human coronavirus HKU1 (HCoV-HKU1)	No Ct	No Ct	25.24	No Ct	No Ct	27.07
Human metapneumovirus (hMPV)	No Ct	No Ct	25.59	No Ct	No Ct	29.73
Parainfluenza virus 1 (PIV-1)	No Ct	No Ct	12.25**	No Ct	No Ct	26.77
Parainfluenza virus 2 (PIV-2)	No Ct	No Ct	12.94**	No Ct	No Ct	26.99
Parainfluenza virus 3 (PIV-3)	No Ct	No Ct	11.23**	No Ct	No Ct	27.50
Parainfluenza virus 4 (PIV-4)	No Ct	No Ct	12.61**	No Ct	No Ct	29.64
Respiratory syncytial virus A (RSV-A)	No Ct	No Ct	13.53**	No Ct	No Ct	26.79
Respiratory syncytial virus B (RSV-B)	No Ct	No Ct	13.96**	–	–	–
Adenovirus	No Ct	No Ct	26.32	No Ct	No Ct	28.47
Influenza B	No Ct	No Ct	13.53**	No Ct	No Ct	29.76
Bocavirus	No Ct	No Ct	26.77	–	–	–
*Haemophilus influenzae*	–	–	–	No Ct***	No Ct	27.51
*Streptococcus pneumoniae*	–	–	–	No Ct	No Ct	27.65
*Streptococcus pyogenes*	–	–	–	No Ct	No Ct	27.94
*Candida albicans*	–	–	–	No Ct	No Ct	27.51
*Pseudomonas aeruginosa*	–	–	–	No Ct***	No Ct	27.57
*Staphylococcus epidermis*	–	–	–	No Ct	No Ct	27.59
*Staphylococcus salivarius*	–	–	–	No Ct***	No Ct	27.46
*Mycoplasma pneumoniae*	–	–	–	No Ct	No Ct	27.21
*Chlamydia pneumoniae*	–	–	–	No Ct	No Ct	27.18
*Legionella pneumophila*	–	–	–	No Ct	No Ct	24.27
*Bordetella pertussis*	No Ct	No Ct	–	No Ct	No Ct	No Ct
*Bordetella parapertussis*	No Ct	No Ct	–	No Ct	No Ct	35.17
*Pneumocystis jirovecii*	No Ct	No Ct	–	No Ct	No Ct	No Ct

The RNA from seven different cell culture-amplified coronaviruses was used to set up the Biomeme SARS-CoV-2 assay on Biomeme Franklin three9 in order to assess the specificity of the test to SARS-CoV-2. Mid-turbinate swabs from common respiratory pathogens were also used to test for cross-reactivity with both the Biomeme and Precision Biomonitoring SARS-CoV-2 tests. Common bacteria were also tested with the Precision Biomonitoring assay, not the Biomeme test.

*According to the Biomeme SARS-CoV-2 test design, the RNA Process Control may not amplify (No Ct) when the two SARS-CoV-2 gene signals are very strong.

**RNA used was previously extracted for use on xTAG RVP FAST v2 (Luminex, Toronto, Canada) or ResPlex II (Qiagen, Hilden, Germany), which both use MS2 as an internal control, hence much lower Ct values.

***Threshold of 500 RFU was not met, Ct value adjusted.

### Reproducibility of the SARS-CoV-2 assays

To assess assay the reproducibility of the Biomeme SARS-CoV-2 test, nucleic acids from eight samples (all at 10^–4^ dilution) were used by three technicians to run the test twice on three different Franklin three9 instruments. Results from this experiment showed similar outcomes regardless of the technician or instruments (n = 144), and the coefficient of variation ranged from 2.7 to 13.6% (Table 3A–C), except in one case (Table 3A, technician 3, second run, RNA Process Control) where 18.4% was calculated. However, this relatively high coefficient of variation did not affect the results as the mean Ct value for that particular run was similar to that of other runs from different technicians. To assess the reproducibility of the Precision Biomonitoring TripleLock assay, cell culture amplified SARS-CoV-2 was spiked into an oropharyngeal sample from a patient and was run in five replicates daily over five days on three different Franklin three9 instruments. Results indicate that all reactions (n = 75) yielded similar results (Table 3D), with the coefficient of variation across thermocyclers and days ranging from 0.3% to 2.1% (Table 3E).

**Table 3:** Repeatability of Biomeme and Precision Biomonitoring TripleLock SARS-CoV-assay.

(A)									
All targets across individual runs, technicians, and Franklin									
Target	Technician 1			Technician 2			Technician 3		
	Ist run	2nd run	1st & 2nd run	Ist run	2nd run	1st & 2nd run	Ist run	2nd run	1st & 2nd run
**Franklin 1**									
Orf1ab gene	30.62 ± 1.1 (9.8%)	31.38 ± 0.9 (8.0%)	31.00 ± 0.7 (8.8%)	27.17 ± 0.5 (5.6%)	26.09 ± 0.6 (7%)	26.90 ± 0.4 (6.4%)	27.01 ± 0.8 (8.4%)	27.83 ± 1.5 (15.7%)	27.42 ± 0.8 (12.3%)
S gene	26.78 ± 0.5 (5.7%)	27.03 ± 1.9 (7.1%)	26.90 ± 0.4 (6.2%)	25.61 ± 0.2 (2.7%)	24.99 ± 0.3 (3.9%)	25.30 ± 0.2 (3.5%)	26.88 ± 0.4 (4.5%)	26.39 ± 0.6 (6.6%)	26.63 ± 0.4 (5.5%)
RNA process control	30.88 ± 1.2 (11.3%)	29.91 ± 1.0 (9.0%)	30.43 ± 0.8 (10.0)	30.70 ± 1.2 (9.9%)	28.35 ± 1.2 (11.8%)	29.53 ± 0.9 (11.0)	30.69 ± 0.9 (8.2%)	29.91 ± 2.0 (18.4%)	30.30 ± 1.1 (13.6)
**Franklin 2**									
Orf1ab gene	29.40 ± 1.2 (11.4%)	29.02 ± 1.0 (9.8%)	29.21 ± 0.8 (10.3%)	25.72 ± 0.5 (6.0%)	25.26 ± 0.7 (7.8%)	25.49 ± 0.4 (6.8%)	26.20 ± 0.8 (9.0%)	26.31 ± 0.6 (6.7%)	26.25 ± 0.5 (7.7%)
S gene	26.22 ± 0.8 (9.0%)	26.48 ± 0.4 (4.6%)	26.35 ± 0.5 (7.0%)	25.46 ± 0.4 (5.0%)	25.02 ± 0.4 (5.0%)	25.24 ± 0.3 (4.9%)	26.69 ± 0.6 (5.9%)	26.14 ± 0.5 (5.0%)	26.41 ± 0.4 (5.4%)
RNA process control	29.54 ± 1.5 (13.2%)	30.31 ± 1.3 (12.5%)	29.95 ± 1.0 (12.4%)	29.55 ± 1.1 (10.2%)	29.03 ± 1.0 (10.2%)	29.29 ± 0.7 (9.9%)	30.71 ± 1.0 (8.3%)	29.39 ± 0.7 (6.7%)	30.05 ± 0.6 (7.7%)
**Franklin 3**									
Orf1ab gene	29.30 ± 1.0 (9.8%)	30.07 ± 0.9 (8.0%)	29.68 ± 0.6 (8.7%)	25.79 ± 0.6 (6.4%)	25.10 ± 0.7 (8.1%)	25.45 ± 0.5 (7.2%)	26.77 ± 0.8 (8.1%)	27.03 ± 0.8 (8.8%)	26.90 ± 0.6 (8.2%)
S gene	26.34 ± 0.6 (6.4%)	26.59 ± 0.6 (6.0%)	26.46 ± 0.4 (6.0%)	26.33 ± 0.7 (8.0%)	25.25 ± 0.5 (6.0%)	25.79 ± 0.5 (7.1%)	26.28 ± 0.5 (5.5%)	26.12 ± 0.6 (6.9%)	26.20 ± 0.4 (6.0%)
RNA process control	30.19 ± 0.9 (7.6%)	30.67 ± 1.4 (12.6%)	30.45 ± 0.8 (10.3%)	30.80 ± 1.2 (11.0%)	28.13 ± 0.8 (8.5%)	29.47 ± 0.8 (10.6%)	29.72 ± 0.8 (7.1%)	30.47 ± 1.3 (11.7%)	30.12 ± 0.7 (9.6%)

(B)									
All targets across the three Franklin									
Target	Franklin 1	Franklin 2	Franklin 3						
Orf1ab gene	28.35 ± 0.5 (11.5%)	26.98 ± 0.4 (10.3%)	27.34 ± 0.4 (10.3%)						
S gene	26.28 ± 0.2 (5.8%)	26.00 ± 0.2 (6.1%)	26.15 ± 0.2 (6.3%)						
RPC	30.09 ± 0.5 (11.4%)	29.75 ± 0.4 (10.1%)	30.00 ± 0.4 (10.1%)						

(C)									
All targets in the three Franklin combined									
Target	Franklin 1, 2, & 3								
Orf1ab gene	27.56 ± 0.2 (10.9%)								
S gene	26.14 ± 0.1 (6.1%)								
RPC	29.94 ± 0.3 (10.4%)								

(D)									
Day		5′UTR gene	E gene	RNaseP					
1	Mean	28.91	26.84	28.91					
	SD	0.528507	0.528507	0.528507					
	SEM	0.141	0.137	0.134					
	CV	1.892	1.975	1.903					
2	Mean	28.67	28.67	28.67					
	SD	0.453346	0.453346	0.453346					
	SEM	0.121	0.023	0.137					
	CV	1.627	0.331	1.945					
3	Mean	29.07	29.07	29.07					
	SD	0.318276	0.318276	0.318276					
	SEM	0.085	0.040	0.136					
	CV	1.133	0.575	1.925					
4	Mean	29.28	29.28	29.28					
	SD	0.450843	0.450843	0.450843					
	SEM	0.120	0.114	0.125					
	CV	1.594	1.636	1.748					
5	Mean	29.19	29.19	29.19					
	SD	0.458395	0.458395	0.458395					
	SEM	0.123	0.086	0.113					
	CV	1.625	1.242	1.570					

(E)									
		Mean	SD	SEM	%CV				
Franklin D13AB6A254B2	5′UTR gene	28.98	0.374797	0.077	1.32				
	E Gene	26.88	0.336693	0.069	1.279				
	RNaseP	27.28	0.472211	0.096	1.766				
Franklin CC92BD2871DB	5′UTR gene	29.06	0.588501	0.12	2.067				
	E Gene	26.95	0.201179	0.041	0.762				
	RNaseP	27.39	0.478864	0.098	1.785				
Franklin C43440902003	5′UTR gene	29.03	0.501677	0.102	1.764				
	E Gene	26.96	0.442525	0.09	1.675				
	RNaseP	27.76	0.492146	0.1	1.81				

The M1 Sample Prep Cartridge Kit for RNA 2.0 was used to separately extract RNA from cell culture-amplified SARS-CoV-2. Following RNA extraction, three technicians ran the Biomeme SARS-CoV-2 Test (twice) with eight extracts of the same concentration on three different Franklin three9. The mean and standard error of the mean (SEM) of the Ct values from the runs, in addition to the coefficient of variation (CV, shown in brackets), are shown. Tables show the individual and combined runs of the three technicians across the three Franklin three9 (A), the combined runs of the three technicians across the three Franklin three9 (B), and the combined runs of the three technicians and the three Franklin three9 (C). Separate extractions were conducted for the Precision Biomonitoring SARS-CoV-2 test on five separate days across three thermocyclers, the variation in Ct values was described across the across days (D) and the three Franklin three9 (E).

### Clinical evaluation of the SARS-CoV-2 assays

We calculated the clinical diagnostic specificity and sensitivity of both assays by testing a cohort of known positive and negative patient oropharyngeal samples. Ninety patient samples were tested with the Biomeme SARS-CoV-2 tests in comparison to the BD Max 5′UTR assay, 41 of those were negative, 48 were positive, and 1 was inconclusive. Our results indicate that the Biomeme SARS-CoV-2 test had a clinical sensitivity of 98% (one sample was inconclusive) and a clinical specificity of 100% (Suppl. Table. 1A and Table 4A). In parallel, 127 patient specimens were tested with the Precision Biomonitoring TripleLock SARS-CoV-2 assay. Sixty-three samples tested positive while 64 samples tested negative, and the results were in agreement with the laboratory-based RRT-PCR assay (CDC EUA assay) confirming 100% clinical sensitivity and specificity (Suppl. Table. 1B,C and Table 4B). Overall, the performance of both tests in a clinical setting was comparable to multiple clinical RT-qPCR tests in use for the current diagnosis of SARS-CoV-2, suggesting both tests provide similar diagnostic accuracy as seen in the laboratory.

**Table 4:** Evaluation of clinical sensitivity and specificity with the mix of known positive and negative clinical samples.

(A)			
		Clinical result	
		Positive	Negative
Biomeme SARS-CoV-2 test result	Positive	48	0
	Negative	1*	41

(B)			
		Clinical result	
		Positive	Negative
Precision Biomonitoring TripleLock SARS-CoV-2 test result	Positive	63	0
	Negative	0	64

Following the evaluation of the clinical performance of the two assays using known positive and negative clinical samples, the clinical sensitivity and specificity of the Biomeme (A) and Precision Biomonitoring TripleLock (B) SARS-CoV-2 assays were calculated.

### Analysis of the compatibility of alternate swab media with M1 sample prep cartridge

**Figure 2:**
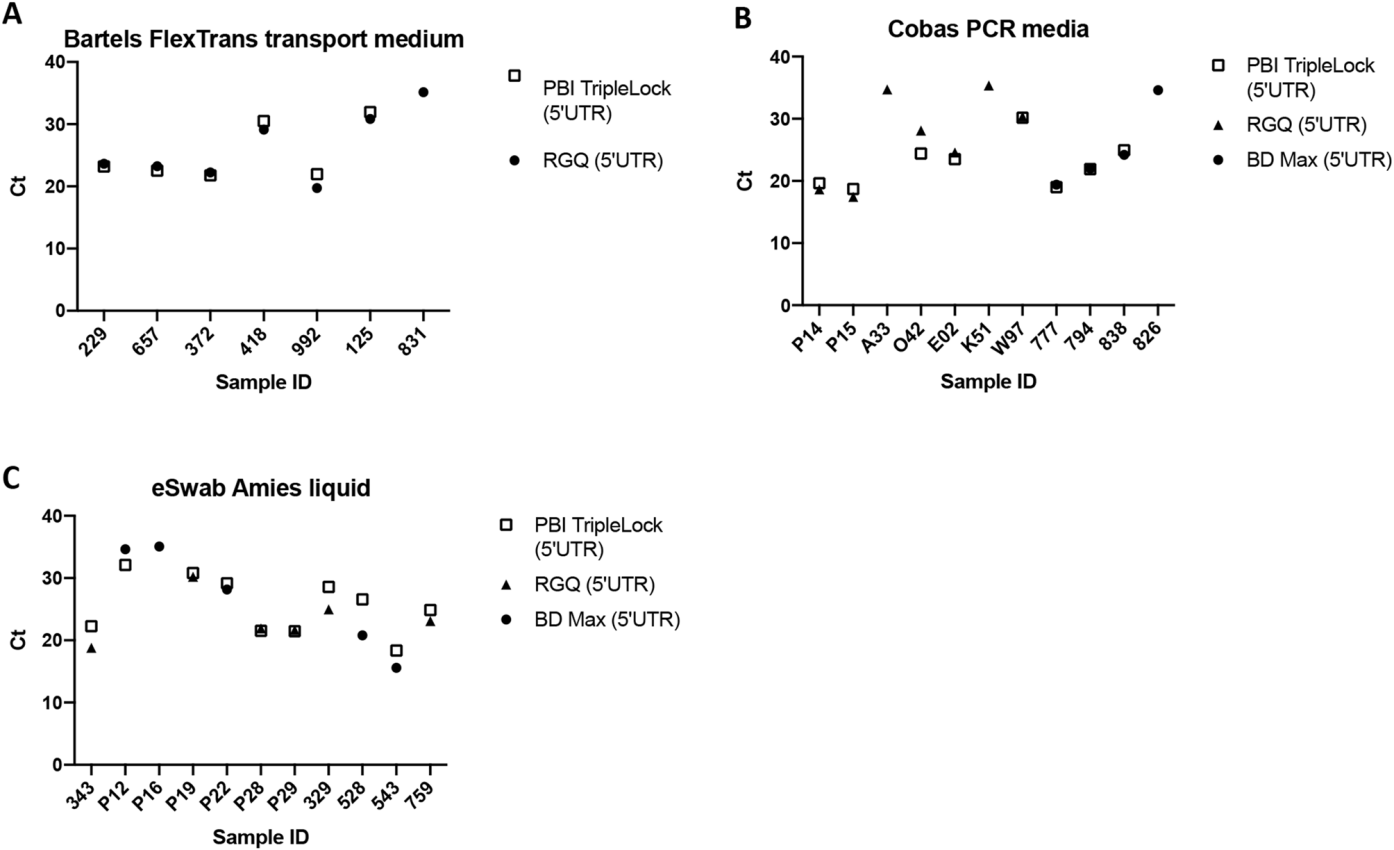
Compatibility of alternate swab media with the point-of-care test. The M1 Sample Prep Cartridge Kit for RNA 2.0 was used to extract 200 ul of swabs sample from Bartels **(A)**, Cobas **(B)**, and eSwabs (≤ 200 µl) **(C)**. SARS-CoV-2 was detected in 6 of 7 Bartels swabs, 10 of 11 e-Swabs, and 8 of 11 Cobas swabs. Paired t-tests comparing the Cq values of the Franklin with the Ct values of the clinical lab test detecting the same targets showed there was no significant difference between the Cq and Ct values for any swab when Ct was detected by the Franklin three9 thermocycler (p_B_ = 0.2864, p_C_ = 0.6566, and p_E_ = 0.0601). *p*_*B*_ p value of Bartels swab; *p*_*C*_ p value of Cobas swab; *p*_*E*_ p value of e-swab swab.

Given the wide variety of sample collection methods, it was necessary to demonstrate the compatibility between the M1 Sample Prep reagents and the most frequently used swab collection media. Following M1 sample extraction, the Precision Biomonitoring TripleLock SARS-CoV-2 assay was able to detect 6 out of 7 (86%) clinical samples collected in Bartels FlexTrans specimen medium, 8 out of 11 (73%) clinical samples in Cobas PCR media, and 10 out of 11 (91%) in Amies liquid (Fig. 2A–C). Specimens that were not detected by the Precision Biomonitoring TripleLock SARS-CoV-2 assay had high Ct values on the BD Max or Rotor Gene Q RT-qPCR platforms used for diagnosis (above 35), suggesting these specimens contained low levels of SARS-CoV-2, perhaps below the LOD of the assay following M1 extraction, or may have suffered sample degradation.

### Evaluation of the SARS-CoV-2 assays with animal samples

To assess the suitability of the Biomeme and Precision Biomonitoring TripleLock SARS-CoV-2 assays for detection of SARS-CoV-2 in animal samples, samples collected from SARS-CoV-2-infected hamsters were tested using these assays. Results indicate that both assays efficiently detected SARS-CoV-2 genetic materials in the nasal wash, rectal, and oral swabs (Table 5A,B) from infected hamsters. These results were in agreement with the laboratory-based RRT-PCR assay used to test the same sample sets.

**Table 5:** Evaluation of clinical performance Biomeme and Precision Biomonitoring TripleLock SARS-CoV-2 assays with hamster samples.

(A)				
Hamster ID	Target (Biomeme)	Ct value		
		Nasal wash	Rectal swab	Oral swab
81	Orf1ab gene	14.42	24.73	32.08
	S gene	14.55	24.83	29.68
	RPC	No Ct*	23.83	24.45
82	Orf1ab gene	12.87	28.25	22.21
	S gene	12.88	29.08	21.12
	RPC	No Ct*	27.46	28.19
83	Orf1ab gene	16.09	26.99	22.59
	S gene	16.23	26.33	21.42
	RPC	No Ct*	25.04	26.43
84	Orf1ab gene	13.29	25.73	20.85
	S gene	13.65	23.17	20.92
	RPC	No Ct*	24.84	No Ct*
85	Orf1ab gene	13.63	23.86	19.88
	S gene	14.05	23.26	19.89
	RPC	No Ct*	23.31	23.28
86	Orf1ab gene	15.61	24.69	20.86
	S gene	16.03	23.22	20.12
	RPC	No Ct*	23.15	19.76
Uninfected_1	Orf1ab gene	–	No Ct	No Ct
	S gene	–	No Ct	No Ct
	RPC	–	28.64	29.51
Uninfected_2	Orf1ab gene	–	No Ct	No Ct
	S gene	–	No Ct	No Ct
	RPC	–	28.85	29.13
Uninfected_3	Orf1ab gene	–	No Ct	No Ct
	S gene	–	No Ct	No Ct
	RPC	–	29.02	30.07

(B)				
Hamster ID	Target (PBI TripleLock)	Ct value		
		Nasal wash	Rectal swab	Oral swab
81	5′UTR gene	16.02	31.49	–
	E_gene	13.22	26.21	–
	RNaseP	No Ct*	39.23	–
82	5′UTR gene	16.10	29.65	23.84
	E_gene	13.15	26.38	20.21
	RNaseP	No Ct*	No Ct*	No Ct*
83	5′UTR gene	17.73	30.62	23.09
	E_gene	14.36	26.26	19.27
	RNaseP	No Ct*	No Ct*	39.81
84	5′UTR gene	16.13	30.03	22.42
	E_gene	11.91	26.50	18.17
	RNaseP	36.33	42.40	35.06
85	5′UTR gene	15.72	25.84	22.99
	E_gene	13.07	23.76	19.18
	RNaseP	No Ct*	33.97	No Ct*
86	5′UTR gene	18.36	23.29	23.39
	E_gene	15.09	22.28	20.95
	RNaseP	No Ct*	31.26	32.24
Uninfected_1	5′UTR gene	–	No Ct	No Ct
	E_gene	–	No Ct	No Ct
	RNaseP	–	39.65	No Ct
Uninfected_2	5′UTR gene	–	No Ct	No Ct
	E_gene	–	No Ct	No Ct
	RNaseP	–	35.07	35.17
Uninfected_3	5′UTR gene	–	No Ct	No Ct
	E_gene	–	No Ct	No Ct
	RNaseP	–	35.24	No Ct

The M1 Sample Prep Cartridge Kit for RNA 2.0 was used to extract RNA from the nasal wash, rectal swab, and oral swab samples from SARS-CoV-2-inoculated hamster on days 2 (#81–83) and 5 (#84–86) post-inoculation. Following RNA extraction, the extracts were tested with Biomeme (A) and Precision Biomonitoring TripleLock SARS-CoV-2 (B) assays on Franklin three9.

*The RNA Process Control may not amplify (No Ct) when the two SARS-CoV-2 gene signals are very strong.

## Discussion

The two SARS-CoV-2 tests evaluated here offer other advantages; the entire kit components and reagents are stable at room temperature, thereby eliminating the complexities associated with handling and storing standard PCR reagents. In addition to being able to complete the extraction protocol in less than 5 min, the M1 extraction cartridge is designed as a single-use cartridge, which helps to eliminate any form of cross-contamination that could occur between samples during nucleic acid extraction. Further, the unique design of the M1 extraction cartridge allows the direct insertion of sample-containing swabs into the lysis buffer compartment of the cartridge. The Franklin three9 features three different channels (green, red, and amber), and allows the viewing of the amplification plots in real-time through a Bluetooth-connected mobile phone display. Overall, the setup is user-friendly and calls the result (positive and negative icons accompanied by amplification plots and Ct values) of the test immediately after the run. However, there is also the option of transferring the data generated from a run to the secure cloud portal (over a wireless connection) where more details about the runs and results are assessed.

There are also limitations to using these tests. Only nine reactions can be performed at a time on the Franklin three9 and the system is not random access; this could be challenging in situations where there are several samples to be tested. Also, basic technical skills (especially pipetting small volumes) are required to successfully run the tests.

During our testing with the animal samples, the internal control (RNaseP) for the Precision Biomonitoring TripleLock assay did not amplify in two oral swab samples from the uninfected hamsters (Table 5B). This result could be because the primers and probes for the RNaseP gene of humans do not uniformly bind and amplify in other animal species, or due to insufficient level of the target in the samples. Based on this information, future iterations of the test would likely include primers and probes for control genes that are present in the target animal species.

In conclusion, the Biomeme SARS-CoV-2 test, the Precision Biomonitoring TripleLock SARS-CoV-2 test, and Franklin three9 offer quick and reliable detection of SARS-CoV-2 in approximately one hour. These observations put the Biomeme and Precision Biomonitoring TripleLock SARS-CoV-2 tests on the map when considering point-of-care diagnostic tests for COVID-19.

## Supplementary Information

Supplementary Table 1.
