# Supplementary material for: Evaluation of mobile real-time polymerase chain reaction tests for the detection of severe acute respiratory syndrome coronavirus 2
Source: Sci Rep. 2021 Apr 30;11:9387. doi: 10.1038/s41598-021-88625-6 (PMC8087814; doi:10.1038/s41598-021-88625-6)
Supplement: Supplementary file 1 — Supplementary Table 1. [file 41598_2021_88625_MOESM1_ESM.pdf]

Evaluation of Mobile Real-Time Polymerase Chain Reaction Tests for the Detection of Severe Acute Respiratory Syndrome Coronavirus 2

Chukwunonso Onyilagha[1*], Henna Mistry[2*], Peter Marszal[1], Mathieu Pinette[1], Darwyn Kobasa[4,6], Nikesh Tailor[4], Yohannes Berhane[1], Charles Nfon[1], Bradley Pickering[1], Samira Mubareka[2], David Bulir[3], Sylvia Chong[3], Robert Kozak[2#] & Aruna Ambagala[1,5]

Supplementary Table 1A

Sample ID	Biomeme SARS-CoV-2 assay Ct values				BD Max 5'UTR assay Ct values			
	Results	Orf1ab	Spike	RPC	Result	5'UTR	E gene	RNaseP
**03806***	?	No Ct*	35.11	23.31	POS	35.09	35.91	24.49
**03821**	POS	17.48	14.33	No Ct	POS	14.84	12.01	24.16
**42356**	POS	28.06	25.68	28.14	POS	26.12	22.83	23.71
**07331**	NEG	No Ct	No Ct	28.06	NEG	No Ct	No Ct	25.82
**01241**	NEG	No Ct	No Ct	28.76	NEG	No Ct	No Ct	25.96
**42404**	POS	29.56	25.77	41.54	POS	23.97	21.94	23.94
**42401**	POS	30.72	26.02	27.73	POS	21.9	No Ct	No Ct
**09451**	NEG	No Ct	No Ct	29.39	NEG	No Ct	No Ct	23.75
**08761**	NEG	No Ct	No Ct	28.44	NEG	No Ct	No Ct	28.22
**42408**	POS	27.31	25.15	29.46	POS	22.91	20.13	26.40
**42403**	POS	22.89	21.76	28.52	POS	21.36	18.87	25.62
**03886**	POS	27.05	24.83	28.10	POS	24.16	21.70	25.64
**04179**	NEG	No Ct	No Ct	29.00	NEG	No Ct	No Ct	24.97
**09281**	NEG	No Ct	No Ct	28.82	NEG	No Ct	No Ct	25.12
**02186**	NEG	No Ct	No Ct	28.28	NEG	No Ct	No Ct	25.11
**42447**	POS	34.26	28.55	27.67	POS	27.06	25.38	27.09
**07387**	NEG	No Ct	No Ct	29.93	NEG	No Ct	No Ct	26.56
**08888**	NEG	No Ct	No Ct	28.84	NEG	No Ct	No Ct	27.16
**01517**	POS	36.70	33.07	27.84	POS	36.15	31.53	24.14
**00389**	NEG	No Ct	No Ct	27.91	NEG	No Ct	No Ct	23.62
**02190**	NEG	No Ct	No Ct	27.95	NEG	No Ct	No Ct	25.42
**42393**	POS	22.33	21.07	31.51	POS	20.36	17.34	23.84
**42444**	POS	29.36	27.94	27.69	POS	34.03	31.50	22.20
**42247**	POS	27.34	26.08	27.72	POS	26.54	23.75	23.52
**42451**	POS	18.36	17.54	No Ct	POS	17.40	14.88	24.42
**42563**	POS	31.51	27.96	27.63	POS	27.18	25.02	25.21
**06480**	NEG	No Ct	No Ct	29.53	NEG	No Ct	No Ct	24.65
**00376**	NEG	No Ct	No Ct	28.99	NEG	No Ct	No Ct	29.78
**42540**	POS	30.35	27.19	27.56	POS	30.70	27.58	22.58
**01173**	NEG	No Ct	No Ct	29.51	NEG	No Ct	No Ct	27.01
**02474**	NEG	No Ct	No Ct	29.22	NEG	No Ct	No Ct	26.45
**05772**	NEG	No Ct	No Ct	28.02	NEG	No Ct	No Ct	24.28
**03988**	POS	30.13	28.14	27.64	POS	29.50	25.83	25.20

**03475**	NEG	No Ct	No Ct	34.67	NEG	No Ct	No Ct	24.27
**42434**	POS	30.43	27.15	31.91	POS	25.05	21.61	No Ct
**09777**	NEG	No Ct	No Ct	32.05	NEG	No Ct	No Ct	24.90
**42645**	POS	32.56	26.34	27.70	POS	26.7	No Ct	No Ct
**42614**	POS	29.82	26.25	28.10	POS	29.52	25.52	23.47
**47770**	NEG	No Ct	No Ct	28.14	NEG	No Ct	No Ct	26.36
**83060**	NEG	No Ct	No Ct	29.48	NEG	No Ct	No Ct	29.96
**50150**	NEG	No Ct	No Ct	35.15	NEG	No Ct	No Ct	26.22
**48940**	NEG	No Ct	No Ct	29.70	NEG	No Ct	No Ct	24.03
**42640**	POS	34.31	29.91	27.78	POS	29.6	No Ct	No Ct
**42712**	POS	18.54	17.38	No Ct	POS	18.49	16.58	25.12
**42697**	POS	30.20	26.93	27.72	POS	28.13	25.61	25.66
**00330**	NEG	No Ct	No Ct	30.59	NEG	No Ct	No Ct	25.90
**07069**	NEG	No Ct	No Ct	32.02	NEG	No Ct	No Ct	31.23
**04259**	NEG	No Ct	No Ct	29.49	NEG	No Ct	No Ct	26.62
**42633**	POS	26.83	25.44	29.01	POS	27.1	No Ct	No Ct
**49758**	NEG	No Ct	No Ct	28.78	NEG	No Ct	No Ct	24.76
**42782**	POS	37.88	34.09	28.04	POS	35.34	33.24	25.00
**42784**	POS	31.51	28.45	27.86	POS	29.75	26.77	24.86
**42631**	POS	22.21	20.93	No Ct	POS	23.28	19.03	24.50
**81257**	NEG	No Ct	No Ct	28.57	NEG	No Ct	No Ct	22.47
**47956**	NEG	No Ct	No Ct	28.57	NEG	No Ct	No Ct	25.57
**47955**	NEG	No Ct	No Ct	29.40	NEG	No Ct	No Ct	24.24
**47953**	NEG	No Ct	No Ct	29.25	NEG	No Ct	No Ct	31.53
**01553**	POS	39.28	35.79	29.58	POS	36.75	34.37	27.63
**02559**	POS	35.58	31.83	28.46	POS	31.87	30.96	28.36
**42809**	POS	35.99	32.71	29.09	POS	33.48	32.61	24.27
**L0001**	POS	32.18	30.57	34.25	POS	30.19	26.45	25.24
**L0004**	POS	20.98	20.14	No Ct	POS	19.56	15.82	29.19
**L0012**	POS	26.34	25.46	31.21	POS	28.13	25.61	25.66
**L0013**	POS	21.75	21.02	No Ct	POS	21.9	No Ct	No Ct
**L0014**	POS	21.91	20.97	No Ct	POS	22.8	No Ct	No Ct
**L0016**	POS	22.12	20.62	No Ct	POS	23.1	No Ct	No Ct
**DI020**	POS	No Ct	37.86	32.21	POS	20.35	19.23	26.65
**DI026**	POS	19.10	18.50	No Ct	POS	17.60	15.87	24.43
**DI002**	POS	23.25	22.08	No Ct	POS	22.05	18.56	No Ct
**DI004**	POS	29.13	28.13	32.48	POS	28.25	24.94	No Ct
**DI010**	POS	25.78	24.85	31.23	POS	25.80	22.36	25.30
**DI011**	POS	36.30	35.16	31.24	POS	34.37	32.99	24.52
**DI013**	POS	17.06	16.06	No Ct	POS	18.34	15.92	No Ct
**DI021**	POS	23.26	22.33	No Ct	POS	23.86	21.34	24.51
**DI022**	POS	29.36	28.44	31.18	POS	29.59	25.17	29.75
**DI025**	POS	29.08	28.19	30.89	POS	31.06	27.76	26.62
**M0002**	POS	22.09	21.04	No Ct	POS	21.02	18.66	No Ct
**M0006**	POS	27.56	26.54	32.13	POS	29.5	No Ct	No Ct
**M0015**	POS	29.83	29.08	31.98	POS	29.28	26.85	26.30
**M0018**	POS	20.34	19.62	No Ct	POS	20.17	17.20	25.08
**RM001**	NEG	No Ct	No Ct	31.70	NEG	No Ct	No Ct	No Ct
**MG001**	NEG	No Ct	No Ct	33.34	NEG	No Ct	No Ct	No Ct
**JS001**	NEG	No Ct	No Ct	33.19	NEG	No Ct	No Ct	No Ct

**MA001**	NEG	No Ct	No Ct	31.19	NEG	No Ct	No Ct	No Ct
**NS001**	NEG	No Ct	No Ct	31.01	NEG	No Ct	No Ct	No Ct
**SL001**	NEG	No Ct	No Ct	32.03	NEG	No Ct	No Ct	No Ct
**VW001**	NEG	No Ct	No Ct	31.86	NEG	No Ct	No Ct	No Ct
**TN001**	NEG	No Ct	No Ct	30.53	NEG	No Ct	No Ct	No Ct
**VM001**	NEG	No Ct	No Ct	31.23	NEG	No Ct	No Ct	No Ct
**LA001**	NEG	No Ct	No Ct	32.05	NEG	No Ct	No Ct	No Ct

Supplementary Table 1B

**Sample ID**	**Precision Biomonitoring TripleLock SARS-CoV-2 assay**				**CDC EUA assay Ct values**			
	**Result**	**E Gene**	**UTR**	**RNase P**	**Result**	**E gene**	**N gene**	**RNaseP**
**24046**	POS	28.8	32.53	22.36	POS	35.02	36.35	22.49
**28902**	POS	30.14	32.46	25.12	POS	33.71	35.47	24.86
**17891**	NEG	No Ct	No Ct	29.09	NEG	No Ct	No Ct	28.84
**25598**	POS	23.95	27.43	24.97	POS	27.96	29.29	26.45
**17790**	NEG	No Ct	No Ct	23.17	NEG	No Ct	No Ct	23.17
**25409**	POS	24.09	27.42	24.12	POS	26.53	28.18	24.39
**26037**	POS	27.15	30.44	27.14	POS	27.99	29.74	26.39
**25631**	POS	28.01	33.51	26.11	POS	31.09	32.8	26.13
**41548**	NEG	No Ct	No Ct	26.1	NEG	No Ct	No Ct	27.51
**17849**	NEG	No Ct	No Ct	26.23	NEG	No Ct	No Ct	25.66
**17996**	NEG	No Ct	No Ct	29.02	NEG	No Ct	No Ct	25.3
**23896**	POS	31.86	34.95	26.22	POS	34.8	37.01	24.93
**26601**	POS	28.58	33.09	23.31	POS	32.12	34.02	23.64
**39659**	NEG	No Ct	No Ct	26.1	NEG	No Ct	No Ct	25.04
**12149**	POS	28.93	31.85	26.14	POS	31.8	33.76	25.06
**25335**	POS	28.06	31.22	26.18	POS	31.24	33.61	25.13
**17301**	NEG	No Ct	No Ct	23.28	NEG	No Ct	No Ct	22.4
**40875**	NEG	No Ct	No Ct	23.17	NEG	No Ct	No Ct	24.18
**27084**	POS	26.77	31.09	25.69	POS	29.17	31.18	23.9
**41067**	NEG	No Ct	No Ct	24.07	NEG	No Ct	No Ct	27.17
**17697**	NEG	No Ct	No Ct	33.47	NEG	No Ct	No Ct	31.13
**24031**	POS	31.16	34.83	28.04	POS	33.73	35.87	27.96
**25185**	POS	23.77	28.6	26.08	POS	26.61	28.11	25.09
**28703**	POS	33.19	38.26	31.92	POS	28.76	30.55	26.52
**28815**	POS	32.85	35.88	26.27	POS	36.16	38.47	24.39
**26140**	POS	20.1	21.01	23.89	POS	18.71	19.58	24.42
**25580**	POS	21.02	23.86	24.11	POS	22.32	23.65	25.35
**46342**	NEG	No Ct	No Ct	28.2	NEG	No Ct	No Ct	26.39

**48456**	NEG	No Ct	No Ct	21.19	NEG	No Ct	No Ct	22.09
**42628**	POS	28.98	31.11	29.07	POS	31.41	33.47	29.58
**46630**	NEG	No Ct	No Ct	25.19	NEG	No Ct	No Ct	25.16
**38958**	POS	23.03	26.11	23.14	POS	22.75	24.14	23.24
**28223**	POS	26.79	29.22	25.2	POS	22.41	24.06	24.68
**48400**	NEG	No Ct	No Ct	26.37	NEG	No Ct	No Ct	23.77
**41536**	POS	23.03	26.31	29.77	POS	24.01	25.31	28.58
**46975**	NEG	No Ct	No Ct	22.21	NEG	No Ct	No Ct	22.13
**47251**	NEG	No Ct	No Ct	27.19	NEG	No Ct	No Ct	25.93
**45099**	NEG	No Ct	No Ct	30.07	NEG	No Ct	No Ct	27.56
**35042**	POS	16.2	18.98	25.67	POS	17.99	18.72	24.27
**41066**	NEG	No Ct	No Ct	26.09	NEG	No Ct	No Ct	24.75
**44934**	NEG	No Ct	No Ct	27.16	NEG	No Ct	No Ct	25.26
**18598**	NEG	No Ct	No Ct	26.06	NEG	No Ct	No Ct	25.7
**34664**	POS	16.19	19.28	21.25	POS	17.49	18.16	22.68
**40492**	POS	22.18	25.34	25.23	POS	23.49	24.52	25.83
**43148**	POS	32.89	31	24.26	POS	32.36	34.08	24.06
**17937**	NEG	No Ct	No Ct	27.04	NEG	No Ct	No Ct	25.97
**47121**	NEG	No Ct	No Ct	24.19	NEG	No Ct	No Ct	23.3
**42731**	NEG	No Ct	No Ct	32.02	NEG	No Ct	No Ct	29.75
**43254**	POS	30.39	31.86	24.3	POS	32.89	34.48	23.17
**42629**	POS	25.96	30.06	25.18	POS	25.39	26.96	25.06
**42653**	NEG	No Ct	No Ct	24.12	NEG	No Ct	No Ct	24.03
**22328**	NEG	41.07	No Ct	29.08	NEG	No Ct	No Ct	27.03
**18454**	NEG	41.81	No Ct	32.21	NEG	No Ct	No Ct	30.38
**39778**	NEG	No Ct	No Ct	28.12	NEG	No Ct	No Ct	25.59
**35286**	POS	27.02	30.03	26.26	POS	24.1	25.41	25.71
**37217**	POS	16.06	19.17	23.16	POS	17.75	18.3	23.24
**39323**	NEG	No Ct	No Ct	24.31	NEG	No Ct	No Ct	23.11
**40949**	POS	24.86	28.73	28.72	POS	26.51	28.16	28.27
**17468**	NEG	No Ct	No Ct	26.15	NEG	No Ct	No Ct	25.45
**17938**	NEG	No Ct	No Ct	29.14	NEG	No Ct	No Ct	29.69
**17532**	NEG	No Ct	No Ct	26.33	NEG	No Ct	No Ct	24.81
**42100**	NEG	No Ct	No Ct	26.26	NEG	No Ct	No Ct	24.81
**40648**	NEG	No Ct	No Ct	23.28	NEG	No Ct	No Ct	23.12
**26982**	POS	29	33.44	27.21	POS	32.59	34.73	26.54
**23982**	POS	18.09	21.36	22.96	POS	20.6	21.8	23.92
**30149**	POS	22.1	25.93	25.09	POS	21.59	22.78	25.28
**17472**	NEG	No Ct	No Ct	32.03	NEG	No Ct	No Ct	30.27

Supplementary Table 1C

Sample ID	Precision Biomonitoring TripleLock SARS-CoV-2 assay				BD Max 5'UTR assay Ct values			
	Result	5'UTR	E gene	RNaseP	Result	5'UTR	E gene	RNaseP
**03806**	POS	31.82	30.48	25.20	POS	35.09	35.91	24.49
**03821**	POS	16.17	13.30	39.58	POS	14.84	12.01	24.16
**42356**	POS	27.08	24.00	25.05	POS	26.12	22.83	23.71
**07331**	NEG	No Ct	No Ct	28.15	NEG	No Ct	No Ct	25.82
**01241**	NEG	No Ct	No Ct	30.08	NEG	No Ct	No Ct	25.96
**42404**	POS	29.77	28.21	24.01	POS	23.97	21.94	23.94
**42401**	POS	29.15	25.15	27.15	POS	21.9	No Ct	No Ct
**09451**	NEG	No Ct	No Ct	29.01	NEG	No Ct	No Ct	23.75
**08761**	NEG	No Ct	No Ct	30.96	NEG	No Ct	No Ct	28.22
**42408**	POS	29.96	25.17	27.30	POS	22.91	20.13	26.40
**42403**	POS	23.98	21.22	26.12	POS	21.36	18.87	25.62
**03886**	POS	29.11	25.04	28.08	POS	24.16	21.70	25.64
**04179**	NEG	No Ct	No Ct	29.21	NEG	No Ct	No Ct	24.97
**09281**	NEG	No Ct	No Ct	30.02	NEG	No Ct	No Ct	25.12
**02186**	NEG	No Ct	No Ct	30.13	NEG	No Ct	No Ct	25.11
**42447**	POS	30.08	27.23	29.24	POS	27.06	25.38	27.09
**07387**	NEG	No Ct	No Ct	30.15	NEG	No Ct	No Ct	26.56
**08888**	NEG	No Ct	No Ct	29.18	NEG	No Ct	No Ct	27.16
**01517**	POS	33.90	30.20	24.26	POS	36.15	31.53	24.14
**00389**	NEG	No Ct	No Ct	27.12	NEG	No Ct	No Ct	23.62
**02190**	NEG	No Ct	No Ct	29.08	NEG	No Ct	No Ct	25.42
**42393**	POS	24.10	20.18	27.26	POS	20.36	17.34	23.84
**42444**	POS	29.36	26.90	22.26	POS	34.03	31.50	22.20
**42247**	POS	28.34	24.62	25.07	POS	26.54	23.75	23.52
**42451**	POS	21.13	17.20	35.89	POS	17.40	14.88	24.42
**42563**	POS	30.01	27.01	26.90	POS	27.18	25.02	25.21
**06480**	NEG	No Ct	No Ct	28.08	NEG	No Ct	No Ct	24.65
**00376**	NEG	No Ct	No Ct	31.12	NEG	No Ct	No Ct	29.78
**42540**	POS	28.35	25.41	23.27	POS	30.70	27.58	22.58
**01173**[#]	NEG	No Ct	0[#]	30.30	NEG	No Ct	No Ct	27.01
**02474**	NEG	No Ct	No Ct	31.04	NEG	No Ct	No Ct	26.45
**05772**	NEG	No Ct	No Ct	27.15	NEG	No Ct	No Ct	24.28
**03988**	POS	31.20	26.85	26.04	POS	29.50	25.83	25.20
**03475**	NEG	No Ct	No Ct	29.15	NEG	No Ct	No Ct	24.27
**42434**	POS	35.22	30.20	26.87	POS	25.05	21.61	No Ct
**09777**	NEG	No Ct	No Ct	29.07	NEG	No Ct	No Ct	24.90
**42645**	POS	28.36	25.10	25.12	POS	26.7	No Ct	No Ct
**42614**	POS	29.99	25.87	24.16	POS	29.52	25.52	23.47
**47770**	NEG	No Ct	No Ct	29.13	NEG	No Ct	No Ct	26.36
**83060**	NEG	No Ct	No Ct	29.47	NEG	No Ct	No Ct	29.96
**50150**	NEG	No Ct	No Ct	30.93	NEG	No Ct	No Ct	26.22

**48940**	NEG	No Ct	No Ct	29.97	NEG	No Ct	No Ct	24.03
**42640**	POS	30.66	28.21	27.18	POS	29.6	No Ct	No Ct
**42712**	POS	20.15	17.15	25.44	POS	18.49	16.58	25.12
**42697**	POS	28.09	25.14	25.19	POS	28.13	25.61	25.66
**00330**	NEG	No Ct	No Ct	29.19	NEG	No Ct	No Ct	25.90
**07069**	NEG	No Ct	No Ct	31.14	NEG	No Ct	No Ct	31.23
**04259**	NEG	No Ct	No Ct	31.05	NEG	No Ct	No Ct	26.62
**42633**	POS	29.17	25.20	27.14	POS	27.1	No Ct	No Ct
**49758**	NEG	No Ct	No Ct	30.26	NEG	No Ct	No Ct	24.76
**42782**	POS	35.36	33.04	29.16	POS	35.34	33.24	25.00
**42784**	POS	30.62	27.30	27.18	POS	29.75	26.77	24.86
**42631**	POS	24.18	20.09	27.17	POS	23.28	19.03	24.50
**81257**	NEG	No Ct	No Ct	27.05	NEG	No Ct	No Ct	22.47
**47956**	NEG	No Ct	No Ct	29.17	NEG	No Ct	No Ct	25.57
**47955**	NEG	No Ct	No Ct	30.13	NEG	No Ct	No Ct	24.24
**47953**	NEG	No Ct	No Ct	31.01	NEG	No Ct	No Ct	31.53
**01553**	POS	35.69	33.51	29.20	POS	36.75	34.37	27.63
**02559**	POS	33.91	31.26	31.10	POS	31.87	30.96	28.36
**42809**	POS	35.32	31.66	27.09	POS	33.48	32.61	24.27

* Inconclusive
#Does not cross 500RFU threshold, adjusted to 0.

Clinical samples with only UTR Ct were run on the BD Max.
Clinical samples with UTR, E gene, and RNaseP were run on Rotor gene Q or CFX96.
